# Public knowledge and awareness of stroke and associated risk factors among adults population in the southern region of Saudi Arabia: A cross-sectional study

**DOI:** 10.1097/MD.0000000000047209

**Published:** 2026-01-16

**Authors:** Shorog Althubait, Rawan M. Alqahtani, Razan M. Alqahtani, Wejdan H.A. Al-Qahtani, Razan H.A. Alqahtani, Taif K. Alasmari, Maha A. Alsharif, Olaa M. Omaish, Thikra K. Alasmari, Reema S. Alqahtani, Syed Esam Mahmood

**Affiliations:** a Stroke Neurologist & Neurointerventionist, College of Medicine, King Khalid University, Abha, Saudi Arabia; b College of Medicine, King Khalid University, Abha, Saudi Arabia; c Department of Family and Community Medicine, College of Medicine, King Khalid University, Abha, Saudi Arabia.

**Keywords:** awareness, knowledge, stroke, stroke prevention

## Abstract

Stroke is a major contributor to long-term disability worldwide. In Saudi Arabia, it ranks as the 3rd leading cause of mortality, necessitating prompt recognition of symptoms and risk factors for timely treatment. This study aims to comprehensively assess stroke knowledge and awareness among the general adult population in the southern region of Saudi Arabia to inform targeted public health interventions for better prevention and care. A cross-sectional study was conducted from June to August 2024, targeting the general adult Saudi population aged 18 years and older residing in the Aseer region. A total of 586 responses were collected through an online questionnaire hosted on Google Forms. The questionnaire included demographic questions and assessed knowledge of stroke symptoms, risk factors, and consequences. Among the 586 participants, 75.6% demonstrated strong general knowledge of stroke. The sample comprised predominantly educated individuals (78.5% with university degrees) and a significant majority of females (74.7%). Recognition of early stroke symptoms was strong, with 65.5% able to identify 5 or more symptoms. Females were significantly more likely than males to identify stroke consequences (*P* = .028). Notably, age (50–70 years) was associated with recognizing symptoms (*P* = .032). However, knowledge gaps were identified regarding risk factors and consequences. Overall knowledge of stroke and its symptoms in the Aseer region is robust. However, targeted public health campaigns are essential to enhance awareness of risk factors and consequences, which may help reduce prehospital delays and improve treatment outcomes.

## 1. Introduction

Stroke is a major global public health concern, ranking as the 2nd leading cause of death and the 3rd leading cause of long-term disability worldwide.^[1]^ In Saudi Arabia, stroke is the 2nd leading cause of mortality, accounting for over 10% of all deaths in the country.^[2]^ As the global population (particularly the elderly) continues to grow, the burden of stroke is rising substantially.^[3]^ The short time frame for administering thrombolytic treatment necessitates immediate hospital admission.^[4]^ However, the majority of stroke patients do not reach the hospital during this critical treatment window. Prompt recognition of stroke symptoms and modifiable risk factors is essential for ensuring timely treatment and reducing stroke-related morbidity and mortality.^[5]^

Previous studies indicate that public knowledge and awareness of stroke symptoms and risk factors remain inadequate, even in high-income countries. Many of these studies have identified factors associated with higher levels of stroke knowledge and awareness, including older age, higher educational attainment, gender, income, and a prior personal or family history of stroke.^[6]^

In a study conducted by Chang Hoon Han et al in 2019 in Korea, it was reported that knowledge about stroke was generally poor, although women demonstrated better awareness than men.^[7]^ Similarly, Dewi Rachmawati et al in 2020 identified educational level as a significant factor influencing stroke knowledge in Indonesia.^[8]^ Another cross-sectional study conducted in Lebanon highlighted that female gender, education level, and employment status were important determinants of stroke-related knowledge.^[9]^ Additionally, a recent study among adults in Taif, Saudi Arabia, revealed that 61.7% of participants were aware of stroke, with younger age and male gender associated with better knowledge. However, this study also indicated gaps in participants’ ability to identify stroke risk factors and symptoms, despite most recognizing stroke as a medical emergency.^[10]^

In a study conducted by Sylvia Saade et al in Lebanon in 2022, it was found that awareness of stroke risk factors and the importance of calling 112 for stroke symptoms was low. Therefore, it is crucial to develop health education programs aimed at reducing stroke morbidity and mortality.^[11]^ Similarly, Adnan Mubaraki et al in 2021 found that while participants acknowledged that stroke constitutes a medical emergency and that timely intervention is vital, there is a significant gap in knowledge regarding the warning signs and symptoms of stroke. Enhancing community awareness of these indicators could potentially improve the speed of patient treatment.^[10]^ Moreover, a study conducted by Reem Alzayer et al in 2023 in Saudi Arabia indicated that participants possess a solid understanding of stroke; however, additional efforts are needed to enhance public awareness and education to improve treatment outcomes.^[12]^

In 2024, Eyman Eltayib et al concluded that stroke knowledge and awareness among the Sudanese population is suboptimal. Additionally, there is a deficiency in early stroke recognition and the adoption of appropriate management strategies, underscoring the necessity for targeted education and awareness campaigns.^[13]^ In the same year, a study by Worawit Wanichanon in southern Thailand revealed that approximately half of the participants were unaware of certain risk factors and warning signs of stroke. Furthermore, most participants exhibited a low to moderate level of awareness and tended to underestimate their stroke risk, even among those with high cardiovascular risk. The FAST acronym may help individuals remember the common warning symptoms of a stroke.^[14]^ Also in 2024, Fatimah Muhanna Alhubail found that the general public in Al-Ahsa exhibited a high level of knowledge regarding stroke symptoms (82.6%), risk factors (90%), and available treatment facilities (80%). Nevertheless, it was evident that the public did not understand the appropriate actions to take in the event of a stroke.^[15]^

Notably, studies conducted in Saudi Arabia have primarily focused on specific populations, such as university students and healthcare patients like diabetic and hypertensive patients, revealing gaps in knowledge regarding stroke risk factors and symptoms.^[16–18]^ Our study primarily aims to fill this gap by providing a comprehensive assessment of stroke knowledge and awareness among the general adult population in the southern region of Saudi Arabia. Secondarily, we aim to assess the understanding of the stroke consequences and the attitude towards stroke. By doing so, we aim to inform targeted public health interventions that can effectively improve stroke prevention and care across the broader community.

## 2. Methodology

A cross-sectional study was conducted in the Aseer region of Saudi Arabia. The targeted population included the general adult population aged 18 years and older residing in the Aseer region. Non-Saudi residents were excluded from the study. The required minimum sample size was determined to be 385 participants, based on a 50% response distribution and a margin of error of ±5%. A total of 586 responses were collected between June 2024 and August 2024.

### 2.1. Participant recruitment

Participants were recruited through online platforms, primarily social media, where the survey link was shared to reach a wider audience. While this method allowed for a diverse range of respondents, we recognize that recruitment via social media may introduce selection bias, as individuals without internet access or those who are less engaged with social media may be underrepresented. Additionally, self-selection bias may occur, as participants who are more interested in health topics may be more likely to respond.

In our research, observer bias was addressed through 2 measures: data collection was conducted by trained researchers using a standardized questionnaire, and assessments were independently verified by a second investigator.

### 2.2. Data collection

The online self-reported questionnaire, adapted from a previous study, was composed of 5 sections covering demographics, general knowledge about stroke, risk factors, symptoms, consequences, and prevention methods. Participants provided informed consent before participating, and ethical approval was obtained from the Research Ethics Committee of King Khalid University (ECM#2024-1607).

### 2.3. Questionnaire development and content

The questionnaire was adapted from previously validated instruments,^[19,20]^ guidelines,^[21]^ and literature^[11–13]^ to ensure a comprehensive assessment of stroke-related knowledge. Specifically, items addressing stroke symptoms were based on the American Heart Association/American Stroke Association guidelines,^[21]^ encompassing both specific symptoms (e.g., sudden weakness, speech difficulties) and nonspecific symptoms (e.g., dizziness, fatigue). This distinction was made to evaluate participants’ ability to recognize symptoms with different clinical implications for early stroke detection and response.

To ensure content validity, the questionnaire incorporated items aligned with standard clinical assessment tools such as the NIH Stroke Scale,^[19]^ which emphasizes key neurological deficits, and the Modified Rankin Scale,^[20]^ which assesses the degree of disability or dependence in daily activities post-stroke. While these scales are primarily used for clinical assessment rather than public knowledge, their core components informed the framing of symptom recognition questions and severity assessments included in the survey.

### 2.4. Validation and pilot testing

The questionnaire underwent a pilot test with 30 participants to assess clarity, relevance, and reliability. Based on feedback, minor modifications were made to improve comprehension. The final version was then used for data collection.

### 2.5. Scoring and knowledge classification

Each correct answer was assigned 1 point, with the total possible score reflecting overall stroke knowledge. Participants scoring above 50% of the total points were classified as having “good knowledge,” consistent with prior studies in similar populations. The differentiation between recognition of specific versus nonspecific symptoms allowed for nuanced analysis of awareness levels and potential impacts on timely stroke recognition.

Participants’ stroke awareness was assessed through a structured questionnaire encompassing knowledge of early stroke symptoms and risk factors. To analyze the data, participants were dichotomized in Table 3 based on their ability to identify at least 1 early stroke symptom and 1 risk factor. This classification was intentionally chosen to represent the minimum level of awareness deemed necessary for effective stroke recognition and prevention. By focusing on the identification of at least 1 symptom and 1 risk factor, the aim was to evaluate a foundational understanding of stroke among participants. Recognizing even a single symptom or risk factor is a critical 1st step that can prompt individuals to seek medical attention and adopt preventive behaviors. Alternative classification approaches, such as requiring recognition of multiple symptoms or risk factors, could have skewed the analysis by potentially excluding individuals who, despite limited knowledge, still possess critical information that may facilitate timely action.

### 2.6. Statistical analysis

Statistical Package for the Social Sciences version 24 for windows (SPSS Inc., Chicago) was used to analyze the responses, demographics, and responses for each section were described as frequencies and percentages. Bivariate analysis was performed using the Chi-square test to evaluate differences between frequencies; however, in cases where expected frequencies were too low, Fisher exact test was used instead. The correct responses for each question were calculated, and a knowledge level above 50% was classified as good knowledge. Multivariate analysis using logistic regression was used to detect the factors associated with the knowledge level considering answering no correct answer as 0, and answer ≥ 1 as 1. *P* values < .05 were considered statistically significant.

## 3. Results

### 3.1. Participants demographics, educational, and marital status

A total of 586 adults participated in this study, of which 581 were Saudi. Table 1 details the sociodemographic characteristics of 586 participants, covering residence, age, gender, profession, education, marital status, nationality, and income. Most participants (89.4%) reside in the Southern region, while smaller proportions are from the Middle (5.6%), Western (3.8%), Eastern (0.9%), and Northern (0.3%) regions. The sample is concentrated in younger age groups, with 41.8% aged 18 to 29 and 41.1% aged 30 to 49, while 17.1% are aged 50 to 70. Gender distribution shows a significant imbalance, with females comprising 74.7% of participants, compared to 25.3% males. Professionally, 41.1% are employed full time, 28.8% are students, 18.3% are unemployed, and smaller groups are retired (8.2%) or working part time (3.6%).

**Table 1:** Sociodemographic characteristics of participants.

Sociodemographics		Frequency	%
Residence	Southern	524	89.4
	Eastern	5	0.9
	Northen	2	0.3
	Western	22	3.8
	Middle	33	5.6
Age	18 to <29	245	41.8
	30–49	241	41.1
	50–70	100	17.1
Gender	Male	148	25.3
	Female	438	74.7
Profession	Student	169	28.8
	Unemployee	107	18.3
	Retired	48	8.2
	Part time	21	3.6
	Full time	241	41.1
Education level	Primary	4	0.7
	Secondary	110	18.8
	Middle	12	2
	University	460	78.5
Marital Status	Widow	8	1.4
	Single	240	41
	Married	326	55.6
	Divorced	12	2
Nationality	Saudi	581	99.1
	Non-Saudi	5	0.9
Income	Middle	455	77.6
	High	55	9.4
	Low	76	13

The education level is notably high, with 78.5% holding a university degree, 18.8% having secondary education, and a very small percentage having primary (0.7%) or middle-level education (2.0%). Marital status reveals that 55.6% of participants are married, 41.0% are single, and smaller percentages are divorced (2.0%) or widowed (1.4%). This breakdown reflects a predominantly Southern, educated, and female gender, with a focus on younger and middle-aged adults, and a diverse range of professional statuses. Finally, in terms of income, the majority of participants (77.6%) report a middle-income level, while smaller proportions identify as having high (9.4%) or low (13.0%) income levels.

### 3.2. Stroke knowledge and awareness

Table 2 presents the number of correct answers given by 586 participants on general stroke knowledge, risk factors, early symptoms, and consequences of stroke. For general stroke knowledge, 1.2% of participants answered 2 questions correctly, while 5.8% answered 3. The largest groups answered 5 (32.8%) and 6 (42.8%) questions correctly, indicating that 75.6% of participants had a strong level of general knowledge. Regarding stroke risk factors, 81.4% of participants could identify at least 1 risk factor. The ability to identify risk factors increased, with 6.8% identifying 4 and 11.8% identifying 6. A notable 33.4% of participants identified 9 risk factors, with 44.7% identifying 6 or more, showing good awareness of stroke risk factors. For early stroke symptoms, 14% could not identify any, while 16% recognized 6, and 35% identified all 7 symptoms. Overall, 65.5% of participants identified 5 or more symptoms, reflecting strong symptom recognition. In terms of stroke consequences, 13.7% could not identify any, while 16.2% identified 4, and 53.9% recognized 5 consequences. This indicates that the majority of participants (53.9%) have a good understanding of stroke consequences, though some had limited knowledge.

**Table 2:** Number of correct answers of general knowledge, identified risk factors, early symptoms, and consequences of the stroke.

(N = 586)		Frequency	%	Cumulative (%)
Number of correct answers regarding stroke in the general knowledge	2	7	1.2	1.2
	3	34	5.8	7.0
	4	102	17.4	24.4
	5	192	32.8	57.2
	6	251	42.8	100.0
Number of identified risk factors of stroke	0	56	9.6	9.6
	1	11	1.9	11.4
	2	17	2.9	14.3
	3	19	3.2	17.6
	4	40	6.8	24.4
	5	50	8.5	32.9
	6	69	11.8	44.7
	7	63	10.8	55.5
	8	65	11.1	66.6
	9	196	33.4	100.0
Number of identified early symptoms of stroke	0	79	14	13.5
	1	11	2	15.4
	2	19	3	18.6
	3	39	7	25.3
	4	56	10	34.8
	5	84	14	49.1
	6	96	16	65.5
	7	202	35	100.0
Number of identified consequences of stroke	0	80	13.7	13.7
	1	18	3.1	16.8
	2	23	3.9	20.7
	3	53	9.0	29.7
	4	95	16.2	46.0
	5	316	53.9	100.0

**Figure 1.**
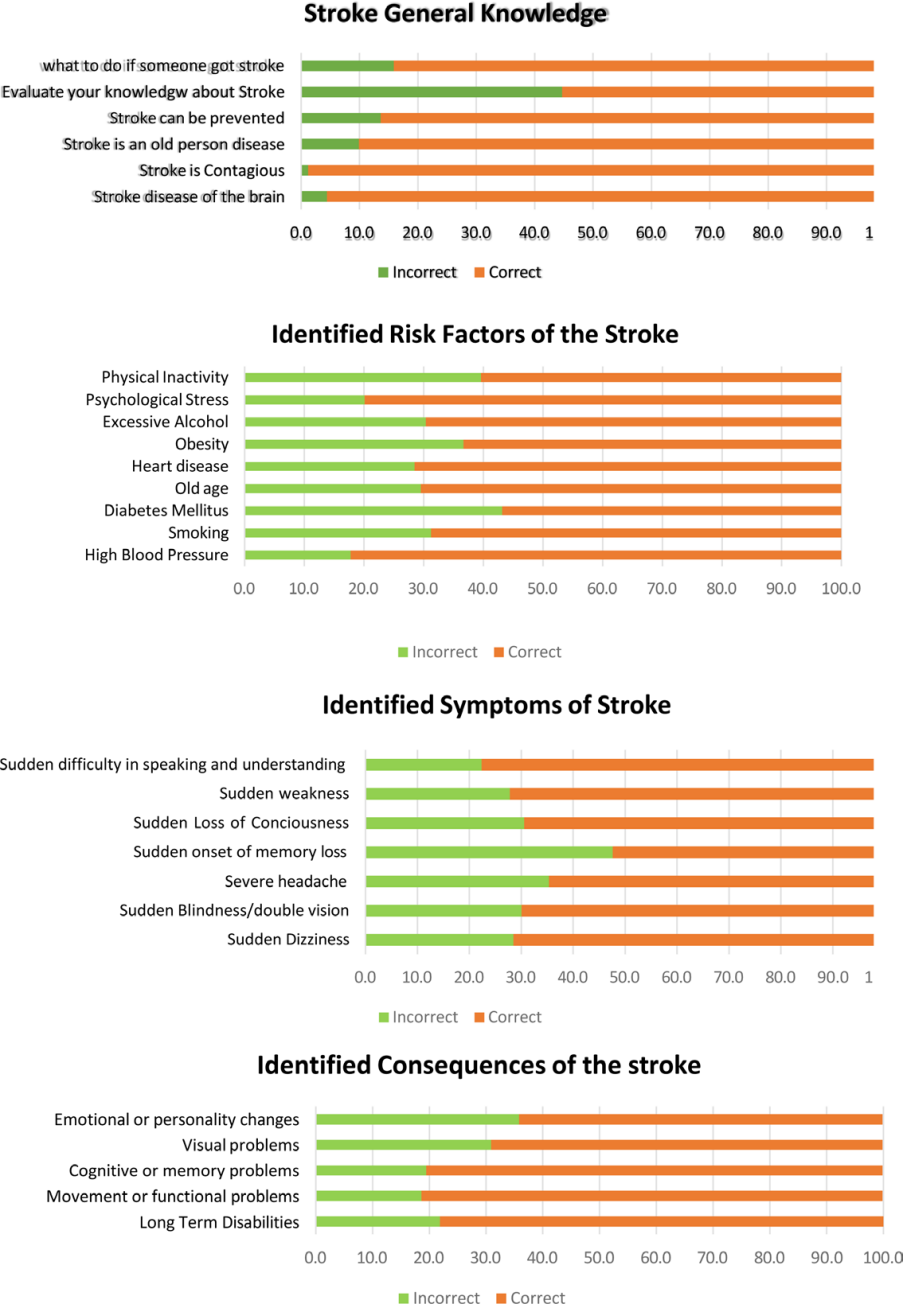
Knowledge regarding stroke.

Figure 1 provides an overview of stroke knowledge, symptoms, risk factors, and consequences among participants. While most correctly identified stroke as a brain disease, many mistakenly believed it to be age-related. Participants demonstrated higher awareness of general symptoms, such as headaches and dizziness, but had lower recognition of critical stroke warning signs, including sudden unconsciousness and speech difficulties. Respondents showed a strong understanding of risk factors like high blood pressure, diabetes, and inactivity, but less awareness of heart disease and the role of alcohol. Stroke consequences were well recognized in terms of disabilities and movement issues, though fewer identified emotional or cognitive changes. Targeted education is needed to address these gaps.

### 3.3. Correlation between stroke knowledge and sociodemographic variables

Table 3 presents a bivariate analysis of the relationship between identifying 1 or more early stroke symptoms and risk factors, considering sociodemographic variables. Most participants, regardless of group, were from the Southern region, with 89.9% identifying early symptoms and 89.3% identifying risk factors. There was no significant association between residence and stroke knowledge (*P* = .919 and 0.63). Age, however, showed a significant association with identifying early stroke symptoms (*P* = .043), as 43.8% of those aged 18 to 29 identified symptoms, compared to 29.1% who did not. No significant association was found between age and identifying risk factors (*P* = .23).

**Table 3:** Bivariate analysis of early symptom(s) identified ≥ 1, risk factor(s) identified.

			Early symptom(s) identified			Risk factor(s) identified		
			≥1			≥1		
			No	Yes	*P* value	No	Yes	*P* value
			(n = 79)	(n = 507)		(n = 35)	(n = 560)	
Residence	Southern	N	71	453	.919	50	474	.63
		%	89.90%	89.30%		89.30%	89.40%	
	Eastern	N	1	4		0	5	
		%	1.30%	0.80%		0%	0.90%	
	Northen	N	0	2		0	2	
		%	0.00%	0.40%		0%	0.40%	
	Western	N	2	20		1	21	
		%	2.50%	3.90%		1.80%	4.00%	
	Middle	N	5	28		5	28	
		%	6.30%	5.50%		8.90%	5.30%	
Age	18 to <29	N	23	222	.043	19	226	.23
		%	29.10%	43.80%		33.90%	42.60%	
	30–49	N	41	200		29	212	
		%	51.90%	39.40%		51.80%	40.00%	
	50–70	N	15	85		8	92	
		%	19.00%	16.80%		14.30%	17.40%	
Gender	Male	N	33	115	<.001	22	126	.01
		%	41.80%	22.70%		39.30%	23.80%	
	Female	N	46	392		34	404	
		%	58.20%	77.30%		60.70%	76.20%	
Profession	Student	N	16	153	.39	11	158	.12
		%	20.30%	30.20%		19.60%	29.80%	
	unemployed	N	18	89		14	93	
		%	22.80%	17.60%		25.00%	17.50%	
	Retired	N	7	41		4	44	
		%	8.90%	8.10%		7.10%	8.30%	
	Part time	N	2	19		4	17	
		%	2.50%	3.70%		7.10%	3.20%	
	Full time	N	36	205		23	218	
		%	45.60%	40.40%		41.10%	41.10%	
Educational level	Primary	N	1	3	.76	0	4	.92
		%	1.30%	0.60%		0%	0.80%	
	Secondary	N	17	93		11	99	
		%	21.50%	18.30%		19.60%	18.70%	
	Middle	N	1	11		1	11	
		%	1.30%	2.20%		1.80%	2.10%	
	University	N	60	400		44	416	
		%	75.90%	78.90%		78.60%	78.50%	
Marital status	Widow	N	1	7	.145	1	7	.12
		%	1.30%	1.40%		1.80%	1.30%	
	Single	N	23	217		16	224	
		%	29.10%	42.80%		28.60%	42.30%	
	Married	N	53	273		39	287	
		%	67.10%	53.80%		69.60%	54.20%	
	Divorced	N	2	10		0	12	
		%	2.50%	2.00%		0.00%	2.30%	
Nationality	Saudi	N	78	503	.52	56	525	.604
		%	98.70%	99.20%		100.00%	99.10%	
	Non-Saudi	N	1	4		0	5	
		%	1.30%	0.80%		0.00%	0.90%	
Income level	Middle	N	56	399	.12	41	414	.09
		%	70.90%	78.70%		73.20%	78.10%	
	High	N	7	48		3	52	
		%	8.90%	9.50%		5.40%	9.80%	
	Low	N	16	60		12	64	
		%	20.30%	11.80%		21.40%	12.10%	

Gender showed a highly significant association with identifying both early symptoms (*P* < .001) and risk factors (*P* = .01), with females more likely to identify both. Males made up 41.8% of those who did not identify early symptoms and 39.3% who did not identify risk factors. No significant associations were found between profession, education level, or marital status and identifying stroke symptoms or risk factors. Most participants held a university degree, were married, and full-time workers predominated. Nationality (mostly Saudi) and income level showed no significant associations with identifying symptoms or risk factors. Middle-income participants predominated across both groups.

### 3.4. Correlation between hospitalization decisions and stroke consequences by sociodemographic factors

Table 4 presents a bivariate analysis of the relationship between taking a stroke patient to the hospital and identifying stroke consequences, with various sociodemographic factors. Participants from the Southern region formed the majority in both categories: 90.3% of those who did not take a patient to the hospital and 89.2% of those who did, with no significant association (*P* = .85 and 0.79). For age, 35.5% of those who did not take a patient to the hospital were aged 18 to 29, compared to 43.0% who did (*P* = .39). Additionally, no significant association was found between age and identifying stroke consequences (*P* = .18). Gender showed a near-significant association with taking a patient to the hospital (*P* = .057), with males making up 18.3% of those who did not, compared to 26.6% who did. Gender was significantly associated with identifying stroke consequences (*P* = .02), with females more likely to identify consequences. Employment, educational level, marital status, nationality, and income level were not significantly associated with either outcome. Full-time workers, university graduates, Saudis, and middle-income participants were the most represented across both groups. These findings indicate that gender plays a role in stroke consequence awareness, but other sociodemographic factors showed no significant associations.

**Table 4:** Bivariate analysis of taking a patient who is experiencing stroke to the hospital and consequences identified (≥1).

			Taking a patient who is experiencing stroke to the hospital			Consequences identified (≥1)		
			No	Yes	*P* value	No	Yes	*P* value
Residence	Southern	N	84	440	.85	71	453	.79
		%	90.3%	89.2%		88.8%	89.5%	
	Eastern	N	1	4		0	5	
		%	1.1%	0.8%		0.0%	1.0%	
	Northen	N	0	2		0	2	
		%	0.0%	0.4%		0.0%	0.4%	
	Western	N	2	20		3	19	
		%	2.2%	4.1%		3.8%	3.8%	
	Middle	N	6	27		6	27	
		%	6.5%	5.5%		7.5%	5.3%	
Age	18–29	N	33	212	.39	35	210	.18
		%	35.5%	43.0%		43.8%	41.5%	
	30–49	N	43	198		37	204	
		%	46.2%	40.2%		46.3%	40.3%	
	50–70	N	17	83		8	92	
		%	18.3%	16.8%		10.0%	18.2%	
Gender	Male	N	17	131	.057	28	120	.02
		%	18.3%	26.6%		35.0%	23.7%	
	Female	N	76	362		52	386	
		%	81.7%	73.4%		65.0%	76.3%	
Profession	Student	N	24	145	.06	19	150	.73
		%	25.8%	29.4%		23.8%	29.6%	
	unemployee	N	18	89		17	90	
		%	19.4%	18.1%		21.3%	17.8%	
	Retired	N	9	39		5	43	
		%	9.7%	7.9%		6.3%	8.5%	
	Part time	N	8	13		3	18	
		%	8.6%	2.6%		3.8%	3.6%	
	Full time	N	34	207		36	205	
		%	36.6%	42.0%		45.0%	40.5%	
Educational level	Primary	N	1	3	.24	0	4	.67
		%	1.1%	0.6%		0.0%	0.8%	
	Secondary	N	24	86		18	92	
		%	25.8%	17.4%		22.5%	18.2%	
	Middle	N	1	11		2	10	
		%	1.1%	2.2%		2.5%	2.0%	
	University	N	67	393		60	400	
		%	72.0%	79.7%		75.0%	79.1%	
Marital status	Widow	N	1	7	.33	0	8	.39
		%	1.1%	1.4%		0.0%	1.6%	
	Single	N	34	206		30	210	
		%	36.6%	41.8%		37.5%	41.5%	
	Married	N	54	272		47	279	
		%	58.1%	55.2%		58.8%	55.1%	
	Divorced	N	4	8		3	9	
		%	4.3%	1.6%		3.8%	1.8%	
Nationality	Saudi	N	93	488	.42	79	502	.52
		%	100.0%	99.0%		98.8%	99.2%	
	Non-Saudi	N	0	5		1	4	
		%	0.0%	1.0%		1.3%	0.8%	
Income level	Middle	N	74	381	.56	57	398	.13
		%	79.6%	77.3%		71.3%	78.7%	
	High	N	6	49		7	48	
		%	6.5%	9.9%		8.8%	9.5%	
	Low	N	13	63		16	60	
		%	14.0%	12.8%		20.0%	11.9%	

### 3.5. Multivariate analysis

For identifying symptoms (≥1), gender was a significant predictor, with females more likely to report at least 1 symptom (OR = 2.942, 95% CI = 1.704–5.081, *P* < .001), using males as the reference category. Other factors such as employment status and age group, with 18 to <29 years as the reference category, were not significantly associated with symptom identification.

In terms of identifying risk factors (≥1), females were also more likely to report at least 1 factor (OR = 2.517, 95% CI = 1.352–4.686, *P* = .004), while employment status and age group did not show significant differences.

Regarding identifying consequences (≥1), females were more likely to identify consequences (OR = 1.822, 95% CI = 1.067–3.111, *P* = .028). Participants aged 50 to 70 years were more likely to identify consequences compared to those aged 18 to <29 (OR = 3.187, 95% CI = 1.105–9.192, *P* = .032). The reference category for age group is 18 to <29 years.

The reference categories in Table 5 are as follows: for gender, males serve as the reference category; for age group, the reference category is 18 to 29 years; and for employment status, students are the reference category.

**Table 5:** Multivariate logistic regression.

	*B*	*P* value	OR	Confidence interval (95%)	
				Lower	Upper
Symptom(s) identified (≥1)					
Constant	1.030	.564	2.802		
Profession					
Unemployed vs student	-0.320	.500	0.726	0.286	1.843
Retired vs student	0.483	.486	1.621	0.417	6.302
Part time vs student	0.751	.397	2.120	0.372	12.069
Full time vs student	0.385	.439	1.470	0.553	3.905
Female vs male	1.079	<.001	2.942	1.704	5.081
Age groups					
30–49 vs 18–29	-0.701	.211	0.496	0.165	1.487
50–70 vs 18–29	-0.620	.350	0.538	0.147	1.972
Factor(s) identified (≥1)					
Constant	2.010	<.001	7.466		
Profession		.192			
Unemployee vs student	-0.787	.116	0.455	0.170	1.216
Retired vs student	-0.122	.879	0.885	0.183	4.269
Part time vs student	-1.061	.147	0.346	0.082	1.454
Full time vs student	-0.056	.915	0.945	0.338	2.645
Female vs male	0.923	.004	2.517	1.352	4.686
Age groups		.543			
30–49 vs 18–29	-0.339	.421	0.712	0.312	1.628
50–70 vs 18–29	0.080	.893	1.083	0.340	3.451
Consequence(s) identified (≥1)					
Constant	1.607	<.001	4.988		
Profession		.464			
Unemployee vs student	-0.724	.072	0.485	0.220	1.067
Retired vs student	-0.786	.261	0.456	0.116	1.791
Part time vs student	-0.661	.358	0.516	0.126	2.118
Full time vs student	-0.618	.130	0.539	0.242	1.200
Female vs male	0.600	.028	1.822	1.067	3.111
Age groups		.099			
30–49 vs 18–29	0.320	.346	1.377	0.708	2.677
50–70 vs 18–29	1.159	.032	3.187	1.105	9.192

## 4. Discussion

A strong understanding of stroke symptoms and modifiable risk factors is essential for ensuring timely treatment and guiding prevention efforts.^[22]^ This study indicates a notable improvement in general stroke knowledge among participants compared to previous studies, where Ahmed Y. Al Ameer et al found that 57.4% of participants demonstrated good stroke knowledge,^[23]^ and Mohamed M. Abdel-Latif et al reported that only 42.6% of the Saudi population had acceptable stroke knowledge.^[24]^ Additionally, Adnan A. Mubaraki et al observed that just 8.7% had good knowledge in Taif city.^[10]^ Our findings suggest that recent health campaigns and educational outreach may have contributed to this significant improvement, highlighting the importance of continued efforts to enhance public awareness about stroke.

Among the participants, a substantial majority had a high level of education, and a significant portion were female. This contrasts with the findings of Rasha Almubark et al, who reported that 87% of Saudis had low general health literacy.^[25]^ Our study reflects a more health-literate and educated sample. Additionally, previous research by Ahmed Y. Al Ameer et al found that individuals with university-level education demonstrated better stroke knowledge, with 59.7% showing awareness compared to 40.5% among those with lower education.^[23]^ This alignment suggests that educational attainment plays a critical role in enhancing stroke knowledge and awareness.

A significant portion of participants demonstrated strong recognition of stroke symptoms, with 65.5% able to identify 5 or more early symptoms. This finding is consistent with research by Lin Pothiban et al, which indicated that individuals with stroke risk factors had better knowledge of stroke symptoms.^[26]^ In contrast, studies in China by Junbo Liang et al reported much lower awareness, with only 6.5% demonstrating good knowledge of warning signs, and another Chinese study indicating that between 29.8% and 59.5% recognized stroke symptoms.^[3]^ These comparisons suggest that our sample exhibits a relatively higher level of awareness regarding stroke symptoms, emphasizing the need for ongoing educational efforts.

A significant proportion of participants, 33.4%, were able to identify 9 stroke risk factors, indicating a strong understanding of these factors. This level of awareness surpasses the 45.81% reported in a recent study for adequate knowledge of stroke risk factors, which also noted that 15.4% of participants had no awareness at all. In China, research by Junbo Liang et al found that only 40.4% had good knowledge of risk factors, with just 7.9% able to identify 3 risk factors.^[3]^ In Morocco, Ahmed Kharbach et al reported that high blood pressure was the most recognized risk factor at 55.7%, followed by stress at 48.8%, while only 37.1% acknowledged prior stroke history.^[27]^ These findings highlight regional disparities in stroke knowledge and underscore the importance of targeted educational initiatives.

Fifty-four percent of participants demonstrated a good understanding of the potential consequences of stroke. Notably, females were significantly more likely than males to identify these consequences, and individuals aged 50 to 70 also showed greater awareness. This trend aligns with several international studies.^[6,10,11]^ Logistic regression analysis indicated that female gender was a significant predictor of recognizing stroke consequences. This disparity may stem from cultural and educational factors prevalent in Saudi Arabia. In the Aseer region, women often take on caregiving roles, which may enhance their awareness of health-related issues, including stroke. Furthermore, the high percentage of female participants with university-level education supports global findings that education enhances health literacy.^[25,26]^ A study in Morocco similarly noted that women with higher education were more likely to recognize stroke symptoms,^[10]^ while research in Indonesia highlighted gender-specific health-seeking behaviors as a contributing factor.^[8]^ In recent years, women’s access to health education programs in Saudi Arabia has improved, possibly contributing to their heightened awareness. Campaigns like the Saudi Ministry of Health’s “Stroke Awareness Week” may have effectively engaged female audiences through tailored messaging in clinics and on social media.^[3]^ In contrast, research in Nigeria and Spain has shown higher awareness among males, likely due to occupational exposure to health campaigns.^[6]^ This divergence underscores the necessity for region-specific interventions.

Most participants recognized diabetes mellitus as a stroke risk factor, with many also identifying physical inactivity. This aligns with recent findings where 61.7% of respondents pointed to physical inactivity and 55.5% recognized hypertension as major risks. In contrast, Antje Sundseth et al in Norway reported that only 43% of participants acknowledged at least 1 risk factor.^[28]^ Another Norwegian study found that 26.6% identified smoking as the most important risk factor.^[29]^ In China, 78.9% considered age, 48.1% recognized smoking, and 82.1% identified hypertension as stroke risk factors.^[3]^ These disparities highlight the importance of culturally tailored awareness campaigns to effectively address stroke risk.

Almost half of the respondents recognized sudden memory loss as a symptom of stroke, and a smaller proportion identified sudden weakness as an early warning sign. These findings contrast with an earlier study where 62.6% identified sudden unilateral weakness as a warning sign, and 20.26% could not recognize any warning signs at all. In Morocco, 37.3% acknowledged sudden weakness of the face, arms, or legs as a main symptom,^[24]^ while in Italy, 68.7% recognized hemiparesis.^[30]^ Overall, these results suggest moderate levels of symptom recognition compared to the higher awareness observed in European studies.

A significant portion of respondents recognized emotional and behavioral changes as major consequences of stroke, highlighting an awareness of the psychosocial impacts. This understanding contrasts with previous studies in Thailand and Norway, which often focused more on physical symptoms and rarely addressed the psychosocial aspects.^[26,29]^ This suggests a more biomedical framing of stroke consequences in those studies.

In contrast to our findings, several studies conducted in different regions of Saudi Arabia reported lower levels of knowledge about stroke. For instance, research by Mohamed M. Abdel-Latif et al indicated that 57.4% of participants had poor knowledge,^[24]^ while Adnan A. Mubaraki et al found that 68.5% had poor knowledge in Taif.^[10]^ Another study in Al-Ahsa revealed that 43.1% of diabetic patients had poor awareness, despite understanding the mechanisms of ischemic stroke.^[31]^ In Majmaah, 56.2% correctly answered risk factor questions, with those aged 45 years or older demonstrating higher levels of adequate knowledge at 63.6%.^[32]^ These discrepancies may be attributed to regional variations, differences in population characteristics, sampling methods, and the influence of recent health interventions. Additionally, the time gaps between earlier and later studies could reflect the effects of increasing stroke prevalence and awareness campaigns in Saudi Arabia.

Our study, along with similar studies assessing public knowledge and awareness of stroke, plays a crucial role in addressing knowledge gaps and misconceptions. This work supports the development of targeted educational strategies aimed at enhancing the recognition of stroke symptoms and the urgency of seeking medical care. Promoting awareness is a key step in reducing treatment delays, ultimately enhancing patient outcomes and optimizing emergency response systems.^[33]^

### 4.1. Limitations

This study has several limitations that should be considered when interpreting the findings. First, the sample was not randomly selected; participants were recruited via social media platforms, which may introduce selection bias. Individuals who engage more with social media may have different health awareness levels compared to those who do not. Additionally, the education level of participants was notably high, with 78.5% holding a university degree. This distribution suggests that our online questionnaire delivery may have favored more educated individuals, potentially underrepresenting those with lower educational attainment. This selection bias is significant, as educational background can influence health literacy and awareness of stroke symptoms and risk factors. Consequently, while our findings provide valuable insights into stroke knowledge in the Aseer region, they may not fully reflect the awareness levels of the broader population.

Moreover, the reliance on self-reported data introduces the potential for self-reporting bias. Participants may have overestimated their knowledge or misreported their awareness of stroke symptoms, risk factors, and consequences, affecting data accuracy. Conducting the survey online may also limit participant diversity; those without internet access or who are less familiar with online platforms may be underrepresented, further skewing results. Lastly, the cross-sectional design restricts our ability to infer causal relationships between the assessed variables, necessitating future longitudinal studies to establish causality more definitively.

### 4.2. Future scope of research

Future studies should aim to include more diverse educational backgrounds to ensure a representative sample, enhancing the generalizability of findings. Additionally, employing mixed methods or alternative data collection strategies could help mitigate self-reporting bias and capture a broader range of perspectives. Investigating the effectiveness of targeted educational interventions on stroke awareness and exploring demographic differences in knowledge can also provide deeper insights. Finally, longitudinal studies are essential to establish causal relationships and assess the long-term impact of improved awareness on stroke prevention behaviors.

## 5. Conclusion

This study highlights that the general knowledge of stroke and its symptoms among participants in the Aseer region is relatively strong. However, there is a clear need for targeted public campaigns to enhance awareness specifically regarding stroke risk factors and potential consequences. By focusing on these areas, we aim to improve overall health literacy, which may contribute to reducing prehospital delays and improving treatment outcomes. Future research should continue to explore the effectiveness of these interventions in raising awareness across diverse populations.

## Author contributions

**Conceptualization:** Shorog Althubait.

**Data curation:** Syed Esam Mahmood.

**Formal analysis:** Syed Esam Mahmood.

**Funding acquisition:** Syed Esam Mahmood.

**Investigation:** Shorog Althubait, Rawan M. Alqahtani, Razan M. Alqahtani, Wejdan H.A. Alqahtani, Razan H.A. Alqahtani, Taif K. Alasmari, Maha A. Alsharif, Olaa M. Omaish, Thikra K. Alasmari, Reema S. Alqahtani.

**Methodology:** Shorog Althubait, Rawan M. Alqahtani, Razan M. Alqahtani, Wejdan H.A. Alqahtani, Razan H.A. Alqahtani, Taif K. Alasmari, Maha A. Alsharif, Olaa M. Omaish, Thikra K. Alasmari, Reema S. Alqahtani, Syed Esam Mahmood.

**Project administration:** Shorog Althubait, Syed Esam Mahmood.

**Resources:** Shorog Althubait.

**Software:** Syed Esam Mahmood.

**Supervision:** Shorog Althubait.

**Validation:** Razan M. Alqahtani, Wejdan H.A. Alqahtani, Razan H.A. AlQahtani, Taif K. Alasmari, Maha A. Alsharif, Olaa M. Omaish, Thikra K. Alasmari, Reema S. Alqahtani.

**Visualization:** Shorog Althubait, Syed Esam Mahmood.

**Writing – original draft:** Shorog Althubait, Rawan M. Alqahtani.

**Writing – review & editing:** Shorog Althubait, Syed Esam Mahmood.

## References

[1] Bakraa R Aldhaheri R Barashid M. Stroke risk factor awareness among populations in Saudi Arabia. Int J Gen Med. 2021;14:4177–82. 34385838 10.2147/IJGM.S325568 PMC8352639

[2] Mahfouz AA Abdelmoneim SA Abdu SMM. Understanding the stroke burden in Saudi Arabia: trends from 1990 to 2019 and forecasting through time series analysis. Neurosciences (Riyadh). 2025;30:49–58. 39800416 10.17712/nsj.2025.1.20240092 PMC11753584

[3] Liang J Luo C Ke S Tung T-H. Stroke related knowledge, prevention practices and associated factors among stroke patients in Taizhou, China. Prev Med Rep. 2023;35:102340. 37576842 10.1016/j.pmedr.2023.102340 PMC10413140

[4] Ma H Campbell BCV Parsons MW. Thrombolysis guided by perfusion imaging up to 9 hours after onset of stroke. N Engl J Med. 2019;380:1814–23. 10.1056/NEJMoa1813046 31067369

[5] Dar NZ Khan SA Ahmad A Maqsood S. Awareness of stroke and health-seeking practices among hypertensive patients in a tertiary care hospital: a cross-sectional survey. Cureus. 2019;11:e4774. 31367493 10.7759/cureus.4774 PMC6666879

[6] Alluqmani MM Almshhen NR Alotaibi RA Aljardi OY Zahid HM. Public awareness of ischemic stroke in Medina city, Kingdom of Saudi Arabia. Neurosciences. 2021;26:134–40. 33551378 10.17712/nsj.2021.2.20200105 PMC8024124

[7] Han CH Kim H Lee S Chung JH. Knowledge and poor understanding factors of stroke and heart attack symptoms. Int J Environ Res Public Health. 2019;16:3665. 31569534 10.3390/ijerph16193665 PMC6801587

[8] Rachmawati D Ningsih DK Andarini S. Factors affecting the knowledge about stroke risks and early symptoms in emergency department East Java – Indonesia. MNJ (Malang Neurology Journal). 2020;6:11–9.

[9] Malaeb D Dia N Haddad C. Factors associated with knowledge and awareness of stroke among the Lebanese population: a cross-sectional study. F1000Res. 2022;11:425–12. 35677174 10.12688/f1000research.108734.1 PMC9160706

[10] Mubaraki AA Alqahtani AS Almalki AA. Public knowledge and awareness of stroke among adult population in Taif city, Saudi Arabia. Neurosciences. 2021;26:339–45. 34663706 10.17712/nsj.2021.4.20210057 PMC9037772

[11] Saade S Hallit S Salameh P Hosseini H. Knowledge and response to stroke among Lebanese adults: a population-based survey. Front Public Health. 2022;10:891073. 35719671 10.3389/fpubh.2022.891073 PMC9203897

[12] Alzayer R Barakat M Jirjees F. Knowledge and awareness of stroke and associated factors in the Saudi general population: a cross-sectional study. Front Neurol. 2023;14:1225980. 37808501 10.3389/fneur.2023.1225980 PMC10552853

[13] Eltayib EM Jirjees F Suliman D. Stroke awareness and knowledge in Sudan: A cross-sectional analysis of public perceptions and understanding. Front Public Health. 2024;12:1362979. 38774053 10.3389/fpubh.2024.1362979 PMC11107802

[14] Wanichanon W Ananchaisarp T Buathong N Choomalee K. Knowledge and attitude towards stroke among the population of one rural community in southern Thailand: a survey. BMJ Open. 2024;14:e080269. 10.1136/bmjopen-2023-080269 PMC10859993 38326263

[15] Alhubail FM Al-Mousa AM Albusaad R. Knowledge of symptoms, risk factors, and treatment centers of stroke among the general population of Al-Ahsa, Saudi Arabia. Ann Afr Med. 2024;23:53–61. 38358172 10.4103/aam.aam_147_23 PMC10922188

[16] Basheikh MA Kobeisy SA Bajammal S. Stroke awareness among university students in Saudi Arabia. Sapporo Med J. 2020;54:1–8.

[17] Alkhalifah KM Hunaif AMA Alghamdi BS. Awareness of stroke risk factors and warning signs among diabetic patients in the Aseer Region, Saudi Arabia: a cross-sectional study. Cureus. 2023;15:e42562. 37637536 10.7759/cureus.42562 PMC10460238

[18] Mersal FM Tork H. Stroke risk perception and its awareness among hypertensive patients in Qassim Region, Saudi Arabia. Majmaah J Health Sci. 2020;8:9–8.

[19] Brott T Adams HP Jr Olinger CP. Measurements of acute cerebral infarction: a clinical examination scale. Stroke. 1989;20:864–70. 2749846 10.1161/01.str.20.7.864

[20] Van Swieten JC Koudstaal PJ Visser MC Schouten HJ van Gijn J. Interobserver agreement for the assessment of handicap in stroke patients. Stroke. 1988;19:604–7. 3363593 10.1161/01.str.19.5.604

[21] Powers WJ Rabinstein AA Ackerson T. Guidelines for the early management of patients with acute ischemic stroke: 2019 update to the 2018 guidelines for the early management of acute ischemic stroke: a guideline for healthcare professionals from the American Heart Association/American Stroke Association. Stroke. 2019;50:e344–418. 31662037 10.1161/STR.0000000000000211

[22] Chakroun-Walha O Samet A Abdallah M B. Stroke knowledge among emergency centre visitors: a cross-sectional multicenter survey. Afr J Emerg Med. 2021;11:37–43. 33318912 10.1016/j.afjem.2020.10.012 PMC7724164

[23] Ameer A Alqarni SAM Alqarni ASS. Knowledge, attitude, and practice of stroke among Saudi population in Balqarn Governorate, Kingdom of Saudi Arabia. Int J Med Dev Countries. 2023;7:xxx–xxx.

[24] Abdel-Latif MM Saad SY. Health literacy among Saudi population: a cross-sectional study. Health Promot Int. 2019;34:123–31. 28973389 10.1093/heapro/dax043

[25] Almubark R Basyouni M Alghanem A. Health literacy in Saudi Arabia: Implications for public health and healthcare access. Pharmacol Res Perspect. 2019;7:e514. 10.1002/prp2.514 PMC6687660 31397117

[26] Pothiban L Khampolsiri T Srirat C. Knowledge and awareness of stroke impacts among Northern Thai population. Pac Rim Int J Nurs Res. 2018;22:e85–94.

[27] Kharbach A Obtel M Achbani A. Level of knowledge on stroke and associated factors: a cross-sectional study at primary health care centers in Morocco. Ann Glob Health. 2020;86:1–8. 32742941 10.5334/aogh.2885 PMC7380055

[28] Sundseth K Faiz KW Rønning OM. Factors related to knowledge of stroke symptoms and risk factors in a Norwegian stroke population. J Stroke Cerebrovasc Dis. 2014;23:1608–14. 10.1016/j.jstrokecerebrovasdis.2014.02.026 24809671

[29] Faiz KW Sundseth K Thommessen B. Patient knowledge on stroke risk factors, symptoms and treatment options. Vasc Health Risk Manag. 2018;14:391–9. 10.2147/VHRM.S152173 PMC5808699 29445287

[30] Baldereschi M Di Carlo A Vaccaro B. Stroke knowledge in Italy. Neurol Sci. 2015;36:347–51. 10.1007/s10072-014-1964-5 25280801

[31] Elshebiny A Almuhanna M AlRamadan M Aldawood M Aljomeah Z. Awareness of stroke risk factors, warning signs, and preventive behavior among diabetic patients in Al-Ahsa, Saudi Arabia. Cureus. 2023;15:e35337. 36974251 10.7759/cureus.35337 PMC10039371

[32] Abdalla SM Mohamed EY Elsabagh HM. Stroke perception and risk factors knowledge within Saudi population. Ethics Med Public Health. 2022;20:100736.

[33] Botelho A Rios J Fidalgo AP Ferreira E Nzwalo H. Organizational factors determining access to reperfusion therapies in ischemic stroke – systematic literature review. Int J Environ Res Public Health. 2022;19:16357. 36498429 10.3390/ijerph192316357 PMC9735885

